# Cognitive processes related to problematic pornography use (PPU): A systematic review of experimental studies

**DOI:** 10.1016/j.abrep.2021.100345

**Published:** 2021-04-07

**Authors:** J. Castro-Calvo, V. Cervigón-Carrasco, R. Ballester-Arnal, C. Giménez-García

**Affiliations:** a Department of Personality, Assessment, and Psychological Treatments, Faculty of Psychology, University of Valencia, Spain; b Department of Basic and Clinical Psychology and Psychobiology, Universitat Jaume I, Spain

**Keywords:** Problematic Pornography Use, Cognitive processes, Systematic review

## Abstract

**Introduction:**

Some people experience symptoms and negative outcomes derived from their persistent, excessive, and problematic engagement in pornography viewing (i.e., Problematic Pornography Use, PPU). Recent theoretical models have turned to different cognitive processes (e.g., inhibitory control, decision making, attentional bias, etc.) to explain the development and maintenance of PPU, but empirical evidence derived from experimental studies is still limited. In this context, the present systematic review aimed to review and compile the evidence around cognitive processes related to PPU.

**Methods:**

A systematic review was performed in accordance with PRISMA guidelines to compile evidence regarding cognitive processes related to PPU. We retained and analyzed 21 experimental studies addressing this topic.

**Results:**

Studies were focused on four cognitive processes: attentional bias, inhibitory control, working memory, and decision making. In brief, PPU is related to (a) attentional biases toward sexual stimuli, (b) deficient inhibitory control (in particular, to problems with motor response inhibition and to shift attention away from irrelevant stimuli), (c) worse performance in tasks assessing working memory, and (d) decision making impairments (in particular, to preferences for short-term small gains rather than long-term large gains, more impulsive choice patterns than non-erotica users, approach tendencies toward sexual stimuli, and inaccuracies when judging the probability and magnitude of potential outcomes under ambiguity).

**Conclusion:**

This systematic review offers a comprehensive overview of the current state of knowledge regarding the cognitive features related to PPU, and points out new areas that warrants further research.

## Highlights

• Some people experience symptoms derived from pornography viewing.
• Cognitive processes may be related to the development of Problematic Pornography Use (PPU).
• We performed a systematic review of 21 studies exploring cognitive processes related to PPU.
• We identified 4 cognitive processes relevant for the development and maintenance of PPU.

## 1 Introduction

The advent of the Internet has dramatically changed the way pornography is consumed (Kohut et al., 2020). Nowadays, multiple devices (e.g., laptops, PCs, tablets, smartphones) allow anonymous and free access to an enormous variety of pornographic contents, from any location and 24/7 (Döring & Mohseni, 2018). As a result, during the last years, we have documented an exponential increase in the number of pornography users. Based on website traffic data, Lewczuk, Wojcik, and Gola (2019) estimated that between 2004 and 2016, the proportion of online pornography users increased by 310%. This figure resonates with that reported by Pornhub on its annual report: between 2013 and 2019, the number of visits registered in this popular pornographic website grew from 14.7 to 42 billion (Pornhub., 2013, Pornhub., 2019). Studies conducted from a person-centered approach estimate that lifetime prevalence of pornography consumption is around 92–98% in men and 50–91% in women (Ballester-Arnal, Castro-Calvo, García-Barba, Ruiz-Palomino, & Gil-Llario, 2021). Compared to data collected a decade ago, lifetime prevalence of pornography use has increased by 41% in men and 55% in women between 18 and 25 years old (Ballester-Arnal, Castro-Calvo, Gil-Llario, & Gil-Juliá, 2016). These figures tend to decline as a function of the timeframe explored: in this line, Grubbs, Kraus, and Perry (2019) found that the prevalence of pornography consumption in a US nationally representative sample decreased from 50% (70% of men; 33% of women) when measured within the last year to 31% (47% and 16% respectively) when assessed within the past month, and to 20% (33% and 8%) when measured within the past week.

There is considerable debate regarding the benefits and potential risks of this increasing ubiquity of pornography, especially in adolescents and young people (for a review, see Döring, 2009). For example, some studies highlight that pornography may be an effective mean to satisfy sexual desire (Daneback, Ševčíková, Mänsson, & Ross, 2013), compensate for the lack of knowledge about sexuality and explore sexuality safely (Smith, 2013), add variety to offline sexual relationships (Daneback, Træen, & Månsson, 2009), distract from boredom and everyday problems (Hald & Malamuth, 2008), or assist in the treatment of certain sexual dysfunctions (Miranda et al., 2019). On the other hand, pornography could also cause a broad range of problems as a consequence of either the ‘types of pornographic contents used’ or ‘the way in which pornography is consumed’ (Owens, Behun, Manning, & Reid, 2012). Mainstream porn is focused on male pleasure, pushes females’ fantasies and desires into the background, and rarely depicts responsible sexual behaviors (such as the use of condom during sexual intercourse) (Gorman, Monk-Turner, & Fish, 2010). Even more worrying, many scholars argue that pornographic material is becoming increasingly degrading and violent toward women (Lykke & Cohen, 2015). Whereas recent studies dispute this ‘accepted wisdom’ (Shor & Seida, 2019), there is consensus around the fact that current pornography (both professional and amateur) tend to depict male sexual dominance (Klaassen & Peter, 2015). As a result, it has been suggested that pornography may negatively impact sexuality by: (a) fostering sexist attitudes and abusive behaviors, (b) facilitating the development of sex risk behaviors (e.g., earlier sexual debut, unprotected sexual intercourse, promiscuity, etc.), (c) creating unrealistic body images and standards of sexual performance, (d) breaking traditional values of monogamy and fidelity; or (e) promoting unusual sexual interests (Braithwaite et al., 2015, Döring, 2009, Stanley et al., 2018). Furthermore, there is a growing body of research indicating that pornography could become problematic if carried out abusively in terms of frequency, severity, and functional impairment. Thus, one of the main risks of pornography use is the possibility of developing symptoms and negative outcomes derived from persistent, excessive, and problematic engagement in this activity (Duffy et al., 2016, Wéry and Billieux, 2017).

It is estimated that between 0.8% and 8% of pornography users display signs and symptoms of Problematic Pornography Use (hereafter, PPU) (Ballester-Arnal et al., 2016, Bőthe et al., 2020, Ross et al., 2012). Central symptoms from PPU include: (a) excessive time and effort spent on watching/searching for pornography; (b) impaired self-control over pornography use; (c) failure to fulfill family, social, or work responsibilities; and (d) persistence in the sexual behavior despite its consequences (Efrati, 2020, Wéry and Billieux, 2017). Inspired by criteria used in Substance Use Disorders (SUDs), some authors also include tolerance, abstinence, and craving as common symptoms among these individuals (Allen et al., 2017, Rosenberg et al., 2014). Nonetheless, the applicability of criteria such as withdrawal and tolerance is still under debate (Starcevic, 2016b). As for its conceptualization and classification, PPU has been considered as a subtype of Hypersexual Disorder (HD; Kafka, 2010), as a form of Sexual Addiction (SA; Rosenberg et al., 2014), or as a manifestation of Compulsive Sexual Behavior Disorder (CSBD; Kraus et al., 2018). As an example of the relevance of PPU in SA, Wéry et al. (2016) found that 90.1% of a sample of 72 self-identified sexual addicts reported PPU as their primary sexual problem. This finding resonates with results from the DSM-5 field trial for HD (Reid et al., 2012), in which 81.1% of a sample of 152 patients seeking treatment for this condition reported PPU as their primary problematic sexual behavior. Conversely, Bőthe et al. (2020) found that individuals categorized as problematic pornography users through a data-driven approach scored systematically higher in a measure of HD; indeed, scores in this scale better discriminated between highly engaged but not problematic and problematic pornography users than any other variable (including the frequency of pornography use). As a result, current trends in out-of-control sexual behaviors consider PPU as a subtype of SA/HD/CSBD (the most prominent indeed) rather than as an independent clinical condition (Gola et al., 2020), and also assume that many patients presenting with SA/HD/CSBD will show PPU as their primary problematic sexual behavior. At a practical level, this means that many patients presenting with PPU will be diagnosed with one of this ‘general’ clinical labels, and PPU will emerge as a specifier within this diagnostic framework.

A large body of literature on the cognitive processes underlying SUDs (Kluwe-Schiavon et al., 2020) and Behavioral Addictions (BAs)^1^ (e.g., gambling [Hønsi, Mentzoni, Molde, & Pallesen, 2013], problematic Internet Use [Ioannidis et al., 2019], gaming disorder [Schiebener & Brand, 2017], or problematic social network use [Wegmann & Brand, 2020]) has provided evidence regarding their relevance in terms of manifestation and severity of these clinical conditions. In the field of SUDs, some of the most influential models (e.g., the dual process theory [Bechara, 2005] or the Incentive-sensitization theory [Robinson & Berridge, 2001]) have turned to different cognitive processes to explain the development and maintenance of addictive behaviors. In the field of BAs, the I-PACE model (Brand, Young, Laier, Wölfling, & Potenza, 2016) has proposed that different cognitive processes (e.g., inhibitory control, decision making, etc.) are central in the development and maintenance of these conditions. In a subsequent development of this model, Brand et al. (2019) suggested that this model may also explain the development and maintenance of PPU. Since PPU is considered as a behavioral specifier for HD (Kafka, 2010), the relevance of cognitive impairments when explaining PPU is also recognized by a recent theoretical model of HD: the Sexhavior cycle (Walton, Cantor, Bhullar, & Lykins, 2017). This model proposes the concept of ‘cognitive abeyance’ to explain some of the neuropsychological features behind HD. Despite the obvious importance of exploring cognitive processes behind PPU, empirical studies addressing this aspect have started to be conducted only within recent years. These preliminary studies have supported the relevance of different cognitive processes when explaining PPU (e.g., Antons & Brand, 2020); however, further research is required to confirm their contribution in the development and maintenance of PPU. Furthermore, a work of review and synthesis of the empirical studies conducted so far is required to gather and analyze all the available evidence on this topic. In this context, the present systematic review aimed to review and compile the evidence around cognitive processes related to PPU. Given that PPU may share parallels with SUDs and other BAs, we focused this review in the four cognitive processes typically related to these conditions: attentional bias, inhibitory control, working memory, and decision making (Wegmann & Brand, 2020).

## 2 Methods

This systematic review was performed in accordance with PRISMA (Preferred Reporting Items for Systematic Reviews and Meta-Analyses) guidelines (Moher et al., 2009). Given the heterogeneity of the studies included in this review, we decided to use a qualitative approach based on the analysis of the core findings in each study (narrative synthesis) (Popay et al., 2006). This methodology is advised when studies included in a review are insufficiently similar to allow for alternative quantitative approaches (e.g., *meta*-analysis) or the review scope dictates the inclusion of a wide range of research designs (both statements are applicable to this review).

### 2.1 Literature review and study selection

A systematic search was used to compile evidence regarding cognitive processes related to PPU. Studies were eligible if they (1) examined a cognitive process through an experimental task and (2) linked the results from this task with an aspect directly or indirectly related to PPU. We included studies establishing the following relationships between a particular cognitive process and PPU: (a) studies comparing certain cognitive processes in subjects with and without PPU; (b) studies comparing certain cognitive processes in subjects with and without SA/HD/CSBD (provided that the study specified PPU as the primary problematic sexual behavior of a large proportion of the sample and/or when certain aspects of pornography consumption –e. g., frequency of pornography use– let to distinguish between groups); (c) studies conducted in community samples correlating certain cognitive process with a direct indicator of PPU (e.g., scores in scales assessing PPU); (d) studies conducted in community samples correlating certain cognitive process with an indirect indicator of PPU (e.g., time online watching pornography, scores in scales assessing out-of-control sexual behaviors, etc.); and (e) studies conducted in clinical or community samples correlating certain cognitive processes with indicators of PPU after the exposure to pornography (e.g., arousability when exposed to pornography, craving after doing so, etc.).

We identified eligible studies by searching for published studies reported in English from 2000 to October 2020, using four academic search engines: PubMed, PsycINFO, Web of Science, and Google Scholar. To identify relevant articles, we used different combinations of the following search terms: “porn*” or “sexually explicit material” or “erotica” or “Internet sex*” AND “cognitive process*” or “executive functions” or “attention* bias*” or “working memory” or “inhibition” or “inhibitory control” or “decision-making”. An asterisk after the search term means that all the terms beginning with that root were included in the study search. To identify additional articles, we conducted a complementary search using keywords such as: “porn* addiction” or “problematic porn* use” or “sex* addiction” or “hypersexual disorder” or “compulsive sexual behavior disorder”. Studies retrieved through the last three terms (SA, HD, and CSBD) included clinical samples of patients reporting PPU as their primary sexual outlet, but also patients reporting other sexual problems (e.g., excessive use of Internet chats or sexual webcams, persistent and uncontrolled extra-marital affairs, habitual solicitation of commercial sex workers, etc.). Following the inclusion criteria, studies assessing clinical samples whose problems were not focused on PPU were excluded from this review.

**Fig. 1:**
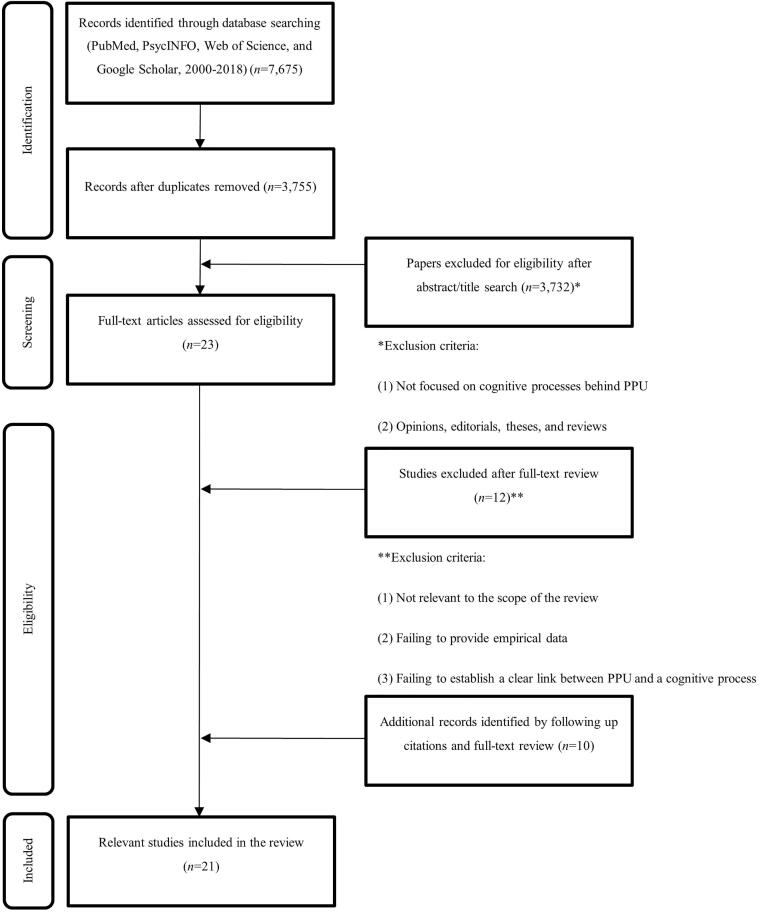
Flowchart of study screening and selection process.

A flowchart detailing the study selection process is shown in Fig. 1. In total, 7,675 studies were identified. After removal of duplicates, we obtained 3,755 records. Two of the review authors (JCC and VCC) examined abstracts and titles for relevant content. Only 23 of these studies were identified as potentially relevant. After a full-text review, we removed 12 of these articles (*n* = 11). To increase the number of studies, we searched the reference list of included articles for relevant literature, identifying 10 additional records that were finally included after a full-text review (*n* = 21).

### 2.2 Data extraction

The following information was extracted from each study (see Table 1). First, we coded the data that were relevant for studies’ identification (*author’s reference* and *date of publication*). We also coded important information for the generalization of the review findings, which included the *country where the study was done* and a *description of the sample* (e.g., size, sex and age distribution, characteristics of the sample, etc.).

**Table 1:** Brief overview of the studies included in this review.

Study identification	Country	Sample description	Cognitive domain	Task/Paradigm	Other measures	Main findings
Kagerer et al. (2014)	Germany	87 heterosexual students: (a) 41 women and (b) 46 men (*M*age = 24.23). Non-clinical sample.	Attentional bias	Dot-probe Task (including both neutral and erotic stimuli); stimuli were presented for 500 ms. Line-orientation Task	Sexual Orientation Questionnaire (SOQ) Sexual Desire Inventory (SDI) Sexual Compulsivity Scale (SCS) Sexual Sensation-Seeking Scale (SSSS)	(1) Sexual sensation seeking was positively correlated with orientating (*r* = 0.33) and negatively correlated with picture categorization (*r* = -0.24). Therefore, sexual sensation seekers tended to answer faster to the dot-probe task when the dot appeared next to a sex picture (compared to a neutral image), and categorize faster pictures depicting sex in the line-orientation task (attentional bias toward sexual stimuli processing). (2) Sexual compulsivity was not significantly correlated with any of the experimental scores, meaning that higher scores in this variable did not facilitate attentional bias toward sexual stimuli.
Doornwaard et al. (2014)	Netherlands	123 participants between 18 and 23 years old (*M*age = 19.99): (a) 61 women and (b) 62 men. Non-clinical sample.	Attentional bias	Dot Probe Task (including both neutral and erotic stimuli); stimuli were presented for 500 ms. Word Search Task	Ad Hoc Questionnaire assessing exposure to online sexual content	(1) Participants who consumed pornography on a regular basis were faster answering to the dot probe task (independently of whether the dot appeared next to a neutral or a sexual picture).
Mechelmans et al. (2014)	United Kingdom	66 heterosexual men: (a) 22 meeting criteria for compulsive sexual behavior (CSB, focusing on compulsive use of online sexually explicit material) (*M*age = 25.14) and (b) 44 healthy controls (*M*age = 24.16).	Attentional bias	Dot Probe Task (including neutral, erotic, and explicit stimuli); stimuli were presented for 150 ms.	Impulsive Behavior Scale (UPPS-P) Beck Depression Inventory (BDI) State-Trait Anxiety Inventory (STAI) The Obsessive-Compulsive Inventory- R Alcohol-Use Disorders Identification Test (AUDIT) Young’s Internet Addiction Test (YIAT) Compulsive Internet Use Scale (CIUS) National Adult Reading Test	(1) Subjects with CSB (PPU as their primary sexual problem) had greater attentional bias to explicit sexual stimuli (pornographic content) (*p* = .022) but not for neutral stimuli (*p* = .495). In particular, subjects with CSB responded faster to the dot-probe task when the dot appeared next to a sexually explicit picture (compared to a neutral image). (2) This attentional bias was observed only when participants were presented with a sexually explicit stimulus; when presented with an erotic stimuli (lower level of explicitness), participants with CSB (PPU as their primary sexual problem) and healthy volunteers responded similarly.
Banca et al. (2016)	United Kingdom	62 heterosexual men: (a) 22 meeting criteria for compulsive sexual behavior (CSB, focusing on compulsive use of online sexually explicit material) (*M*age = 25.14) and (b) 40 healthy controls (*M*age = 25.20).	Attentional bias	Dot Probe Task (including neutral, erotic, and explicit stimuli); stimuli were presented for 150 ms.	Conditioning task Novelty preference task	(1) Subjects having a greater preference for conditioned sexual stimuli (mainly, sexually compulsive with PPU) also showed enhanced attentional bias for sexual stimuli (*p* = .044). (2) In contrast, the preference for novel vs. familiar stimuli was not associated with attentional bias for sexual stimuli (*p* = .458). (3) Important remark: This research reanalyzed data from the study by Mechelmans et al. (2014). Therefore, congruence between both studies are largely due to this overlap. The rationale behind including the study by Banca et al. (2016) as well is that it provides additional insights into the relationship between attentional bias and other neuropsychological and phenomenological features of CSB.
Pekal et al. (2018)	Germany	174 participants: (a) 87 women and (b) 87 men. Participants ranged in age between 18 and 52 years (*M*age = 23.59) 8.9% of male participants and 2.2% of female tested positive for excessive and problematic pornography viewing.	Attentional bias	Visual Probe Task (including both neutral and erotic stimuli); stimuli were presented for 200 or 2,000 ms.	Short-version of the Internet Addiction Test adapted to Internet sex-(s-IATsex). Sexual arousal and craving ratings (i.e., subjective sexual arousal and need to masturbate after being exposed to pornographic stimuli)	(1) Attentional bias toward sexual stimuli (i.e., faster responses to the visual probe task when the arrow appeared next to the sexual stimuli) was correlated with severity of pornography addiction (*r* = 0.23), craving (i.e., desire to masturbate) (*r* between 0.18 and 0.35), and subjective sexual arousal (*r* between 0.11 and 0.25). (2) The relationship between attentional bias toward sexual stimuli and severity of pornography addiction was consistent in both males and females. (3) The relationship between attentional bias toward sexual stimuli and severity of pornography addiction was partially mediated by craving and subjective sexual arousal.
Seok and Sohn (2018)	South Korea	45 heterosexual males (pornography users): (a) 23 meeting criteria for the diagnosis of hypersexual disorder (*M*age = 26.12; *SD* = 4.11) and (b) 22 healthy controls (*M*age = 26.27; *SD* = 3.39). Weekly pornography use: 5.23 times in participants with hypersexuality and 1.80 in healthy men (p < .001; *d* = 3.2).	Inhibitory control (in particular, attentional inhibitory control).	Stroop Task	Sexual Addiction Screening Test-R (SAST-R) Hypersexual Behavior Inventory (HBI) EPI-BOLD: blood oxygen level-dependent responses	(1) Individuals with hypersexual disorder and healthy controls showed similar reaction times when answering to both congruent and incongruent stroop trials. (2) Individuals with hypersexual disorder were less accurate than healthy controls when answering to incongruent stroop trials (82% vs. 89%; *p* < .05), but not when answering to congruent stroop trials. This means that patients with hypersexuality only tend to experience problems in conditions requiring to ignore inappropriate incongruent information.
Seok and Sohn (2020)	South Korea	60 male participants (pornography users): (a) 30 meeting criteria for the diagnosis of problematic hypersexuality (*M*age = 28.81) and (b) 30 healthy men (*M*age = 27.41). Weekly pornography use: 5.23 times in participants with hypersexuality and 1.80 in healthy men (p < .001; *d* = 3.2).	Inhibitory control (in particular, motor inhibitory control).	Go/No-Go Task(using only neutral stimuli -letters- but presented in a neutral or a sexual background)	Functional MRI Sexual Addiction Screening Test (SAST-R) Hypersexual Behavior Inventory (HBI) Barrat Impulsiveness Scale (BIS) Beck Depression Inventory (BDI)	(1) Hypersexual participants performed worse in the Go/No-Go Task (i.e., made more omission/commission) than healthy controls. (2) Differences between participants with hypersexuality and healthy controls are more prominent in no-go trials (trials in which participants should inhibit responses) and when the Go/No-Go task was presented together with a sexual image in the background (compared to a neutral background). (3) As for reaction times, hypersexual individuals responded slower on go trials when sexual background were present (*p* < .05).
Antons and Brand (2020)	Germany	28 heterosexual males pornography users (*M*age = 29.28; *SD* = 8.81): (a) 10 unproblematic pornography users, (b) 9 problematic, and (c) 9 pathological users.	Inhibitory control (in particular, pre-potent motor inhibitory control).	Stop-Signal Task (using neutral stimuli -different coloured dashes- to indicate the kind of trial, and both neutral and pornographic stimuli as background conditions)	Short Internet Addiction Test modified for Internet Pornography (s-IATporn) Hypersexual Behavior Inventory (HBI) Barrat Impulsiveness Scale (BIS-15) Functional MRI	(1) Severity of Internet pornography use (s-IATporn) correlated with reaction times during stop-signal trials in both the neutral (*r* = -0.49) and the pornographic (*r* = -0.52) conditions. In particular, increased severity of Internet pornography use was associated with faster reaction times during stop-signal trials (i.e., better inhibitory control). (2) Craving (i.e., the strong desire to use pornography) correlated with reaction times during stop-signal trials but only in the pornographic condition (*r* = -0.55). Once again, increased craving was associated with faster reaction times during stop-signal trials (i.e., better inhibitory control).
Wang and Dai (2020)	China	70 heterosexual males: (a) 36 with tendency towards cybersex addiction (TCA) (*M*age = 19.75) and (b) 34 healthy controls (HC). (*M*age = 19.76) Weekly pornography use: 3.92 times in individuals with TCA and 1.09 in HC	Inhibitory control (in particular, motor inhibitory control and subsequent motor execution).	Two-Choice Oddball Paradigm (including neutral and pornographic stimuli)	Problematic Internet Pornography Use Scale (PIPUS) Barrat Impulsiveness Scale (BIS-11) *Ad hoc* scale measuring different aspects of cybersex consumption Self-Rating Anxiety Scale (SAS) Self-Rating Depression Scale (SDS) Electroencephalography (EEG)	(1) Both participants with TCA and HC showed slower reaction times when answering to the Two-Choice Oddball Paradigm when it came to sexual stimuli (compared to the neutral stimuli); however, differences in the reaction time between both types of stimuli were more pronounced in patients with TCA. That is, individuals with TCA experienced a poorer inhibitory control when facing sexual stimuli compared to HC.
Laier et al. (2013)	Germany	28 heterosexual males (*M*age = 26.21; SD = 5.95)	Working Memory	*n*-Back Task (4-Back Task using pornographic pictures as stimuli)	Sexual arousal and craving ratings (i.e., subjective sexual arousal and need to masturbate after being exposed to pornographic stimuli)	(1) Performance in the 4-back task (pornographic condition) correlated with indicators of sexual arousal and craving. In particular, subjective sexual arousal after seeing pornographic pictures correlated with the proportion of skips (*r* = 0.45), and craving correlated with proportion of false alarms (*r* = 0.45) (in both cases, indicators of poor performance). This means that individuals showing an increased sexual response to pornography tend to perform worse in the working memory task. (2) General performance in the 4-back test was significantly predicted (*R*^2^ = 27%) by the interaction between sexual arousal and craving after being exposed to sexual stimuli: in particular, participants showing a high level of craving and sexual arousal after being exposed to porn performed worse in the 4-back test.
Au and Tang (2019)	China	Study 1: 24 heterosexual males between 19 and 27 years old (*M*age = 23.08; *SD* = 2.22). Study 2: 27 heterosexual males between 18 and 31 years old (*M*age = 23.0; *SD* = 3.15)	Working memory	Study 1: *n*-Back Task (3-Back Task using letters as stimuli) after the induction of positive, negative, sexual, or neutral emotional states using videoclips. Study 2: *n*-Back Task (3-Back Task using letters, colored circles, or pornographic pictures as stimuli) after the induction of sexual arousal.	Compulsive Sexual Behavior Inventory (CSBI) Discrete Emotions Questionnaire (DEQ) Sexual urge and desire to masturbate after the exposition to pornographic contents, assessed by an *ad hoc* Visual Analogue Scale (VAS) Physiological measures (blood pressure, heart rate, and temperature)	Study1:(1) Participants scoring higher in the CSBI showed a reduced accuracy when answering the 3-back test under the four conditions (*r*_neutal_ = 0.52; *r*_positve_ = 0.72; *r*_negative_ = 0.75; *r*_sexual_ = 0.77). Similarly, high scores in the CSBI correlated with the reaction time when answering the 3-back test under two conditions (*r*_neutal_ = 0.42; *r*_sexual_ = 0.41). In brief, individuals with higher scores in the CSBI tended to perform worse in working memory (less precision an increased time to answer) independently of the emotional condition. Study 2:(2) Participants scoring higher in the CSBI showed a reduced accuracy when answering the 3-back test using different stimuli (*r*_pornography_ = 0.50; *r*_letters_ = 0.45; *r*_circles_ = 0.53). Similarly, high scores in the CSBI correlated with the reaction time when answering the 3-back test using colored circles as stimuli (*r* = 0.39). In brief, individuals with higher scores in the CSBI tended to perform worse in working memory (less precision and increase time to answer) independently of the type of stimuli employed in the 3-back test.
Sinke et al. (2020)	Germany	69 heterosexual males: (a) 38 meeting criteria for the diagnosis of Compulsive Sexual Behavior disorder (*M*age = 36.3; *SD* = 11.2) and (b) 31 healthy controls (*M*age = 37.6; *SD* = 11.7). Weekly pornography use: 213 min per week in participants with CSBD vs. 49 in healthy controls (p < .0.001; d = 0.92).	Working memory	*n*-Back Task (1-Back and 2-Back tasks using letters) with pornographic and neutral pictures in the background	Hypersexual Behavior Inventory (HBI) Revised version of Sexual Addiction Screening Test (SAST-R) Semi-estructured Interview assessing sexual characteristics Sexual Inhibition and Excitation Scales (SIS/SES)	(1) Patients and healthy controls did not differ in their performance in the 1-Back and 2-Back Tasks (accuracy and reaction time) when the tasks were conducted with a neutral picture in the background. (2) When the 1-Back and 2-Back Tasks were conducted with a sexual picture in the background, patients and healthy controls showed significant differences (*p* between 0.01 and 0.03) in terms of accuracy and reaction time: in particular, patients were less accurate (93.4% vs. 97.7% in the 1-Back task; 80.1% vs. 88.2% in the 2-Back task) and showed increased reaction times (668 ms vs. 607 ms in the 1-Back task; 727 ms vs. 696 ms in the 2-Back task). (3) On the contrary, sexually compulsive patients performed better than healthy controls in a task measuring the recognition of sexual stimuli 1 h later of the 1-Back and 2-Back tasks (65.5% vs. 48.3% and 52% vs. 40%). This effect was not observed for neutral stimuli. This suggest that patients with CSBD have a better memorization and recall of pornographic cues, but not for non-sexual stimuli (i.e., better long-term memory and recall of specific sexual stimuli).
Lawyer (2008)	USA	71 participants: (a) 38 men and (b) 33 women between 18 and 57 years old (*M*age = 23.4; *SD* = 7.7). 60% of male participants and 39.5% of female participants were classified as erotica users (i.e., users of erotica in the past and interested in watching erotica in the future)	Decision-making (in particular, delay discounting)	Delay and Probability Discounting Tasks (one assessing discounting for money, the other assessing discounting for erotica).	The Sexual Opinion Survey (SOS) The Sexual Compulsivity Scale (SCS) The Sexual Inhibition/ Sexual Excitation Test (SIS/SES) The Erotica Consumption Scale (ECS)	(1) In both the monetary and the erotica discounting tasks, erotica users preferred smaller reinforcers available immediately than larger reinforcers provided after some delay. Similarly, erotica users preferred small but certain outcomes than larger but uncertain outcomes. (2) In the erotica discounting task, non-erotica users tended to value lower probability and larger delayed outcomes more than higher probability and more immediate outcomes, suggesting that erotica outcomes were aversive to these participants. (3) Two parameters of the erotica discounting tasks were significantly correlated with the SCS (*r* = -0.41). and the SOS (*r* = 0.38). These results indicate that sexual compulsivity was associated with more impulsive choice patterns. Surprisingly, erotophilia significantly correlated with a more reflexive choice pattern (meaning that erotophilic individuals tended to prefer larger delayed outcomes).
Laier et al. (2014)	Germany	82 heterosexual men between 18 and 54 years (*M*age = 25.21; *SD* = 6.23). Participants were cybersex users and spend around 1.4 h per week online for sexual purposes (*SD* = 1.30).	Decision-making (in particular, decision making under ambiguity)	Iowa Gambling Test (IGT) (using pornographic and neutral pictures as stimuli)	Sexual arousal ratings before and after being exposed to pornographic stimuli. Short-version of the Internet Addiction Test adapted to Internet sex-(s-IATsex). *Ad hoc* questionnaire assessing different aspects of cybersex use	(1) Performance on the Iowa Gambling Test was better when sexual stimuli were associated with advantageous decisions and worse when associated with disadvantageous decisions (*d* = 0.69). This means that sexual stimuli may guide the adoption of an advantageous vs. a disadvantageous approach when making decisions under ambiguity. (2) This effect depended on participants’ tendency to get aroused when exposed to sexual stimuli. In individuals reporting low sexual excitation after being exposed to sexual stimuli, whether sexual stimuli were related to advantageous or disadvantageous decisions did not modulate performance on the Iowa Gambling Test. However, in individuals reporting high sexual arousal after sexual picture presentation, performance on the Iowa Gambling Test was worse when sexual pictures were associated with disadvantageous decisions and better when the linked to advantageous decisions.
Mulhauser et al. (2014)	USA	62 male participants: (a) 18 patients between 18 and 68 years old (*M*age = 43.22; SD = 14.52) meeting criteria for hypersexual disorder and (b) 44 healthy control between 18 and 44 years old (*M*age = 21.23; *SD* = 4.55) All the hypersexual subjects (100%) reported PPU as their primary sexual problem.	Decision-making (in particular, decision making under ambiguity)	Iowa Gambling Test (IGT)	Hypersexual Behavior Inventory (HBI) Barrat Impulsiveness Scale (BIS)	(1) Hypersexual patients (PPU as their primary sexual problem) were more likely to select decks with frequent loss penalties than healthy controls (*p* = .047), a pattern of response that leads to a poor performance on the Iowa Gambling Test. (2) General remark: Preference of hypersexual patients for this pattern of response indicates impaired decision-making capabilities and, at a higher-order level, impaired executive functions.
Schiebener et al. (2015)	Germany	104 heterosexual males between 18 and 50 years old (*M*age = 24.29). Non-clinical sample.	Decision-making (in particular, goal-oriented multitasking and self-regulation of behavior)	Balanced Switching Task Porn (BSTporn).	Brief Symptom Inventory (BSI). Short-version of the Internet Addiction Test adapted to Internet sex-(s-IATsex).	(1) Positive correlation between the BSTporn multitasking imbalance (reduction in task performance due to the investment of too much time [overuse] or too little time [neglect] working on pornographic stimuli) and the s-IATsex score (r = 0.28). (2) The BSTporn multitasking imbalance explained 6% of the variance of the s-IATsex test. (3) Participants who obtained higher scores on the s-IATsex tended to overuse or neglect working on pornographic stimuli (i.e., to show a less balanced performance on the cognitive task). (4) General remark: Exposition to pornographic content in people who show tendencies towards cybersex addiction is related to executive control problems in multitasking situations.
Snagowski and Brand (2015)	Germany	123 heterosexual males (*M*age = 23.79; *SD* = 5.10). All the participants were pornography users.	Decision-making (in particular, approach-avoidance tendencies)	Approach-Avoidance Task (AAT) including both neutral and sexual stimuli. Task-relevant instructions (pull or push the stimuli according to their content –sexual vs. neutral–).	Sexual arousal ratings and need to masturbate in front of pornographic stimuli. Short-version of the Internet Addiction Test adapted to Internet sex-(s-IATsex). Hypersexual Behavior Inventory (HBI) Sexual Excitation Scale (SES)	(1) The total reaction time when answering the Approach-Avoidance Task (i.e., indirect measure of attentional biases toward pornographic stimuli) correlated with the HBI (*r*_total score_ = 0.21; *r*_loss of control_ = 0.21; *r*_consequences_ = 0.26), the SES (*r* = 0.26), the level of sexual arousal in front of pornographic stimuli (*r* = 0.25) and the desire to masturbate (*r* = 0.39). (2) The relationship between the level of severity of pornography consumption (i.e., the s-IATsex score) and approach-avoidance tendencies was curvilinear: i.e., individuals with higher scores in the s-IATsex tended to show either extreme approach or extreme avoidance tendencies toward pornographic stimuli. (3) Finally, the relationship between the level of severity of pornography consumption and approach-avoidance tendencies was moderated by the HBI and SES: both approach and avoidance tendencies, when accompanied by high levels of sexual excitation and hypersexuality, resulted in an increased severity of pornography consumption.
Negash et al. (2016)	USA	Study 1:123 undergraduate students between 18 and 27 years old (*M*age = 20): (a) 32 men and (b) 91 women. Study 2:37 undergraduate students between 18 and 28 years old (*M*age = 19): (a) 24 men and (b) 13 women.	Decision-making (in particular, delay discounting)	Delay Discounting Tasks (assessing discounting for money).	*Ad hoc* question assessing frequency of pornography use	Study 1:(1) Frequency of pornography consumption in time 1 predicted delay discounting four weeks later (*β* = 0.21; *p* < .05; *R*^2^ = 19%). That is, participants reporting viewing more pornography demonstrated higher discounting of future rewards (i.e., preference for smaller immediate rewards rather than larger delayed rewards) four weeks later. Study 2:(2) After refraining from pornography consumption for 21 days, participants reported reduced levels of delay discounting (i.e., showed an increase in their preferences for delayed longer gains). This change was larger than that observed for participants refraining from their preferred food, meaning that positive effects of exercising self-control on delay discounting was greater when the refrained appetitive behavior was pornography.
Sklenarik et al. (2019)	USA	58 undergraduate males self-identified as pornography users (*M*age = 19.5; *SD* = 2.4). Four participants were classified as problematic pornography users.	Decision-making (in particular, approach-avoidance tendencies)	Approach-Avoidance Task (AAT) including both neutral and sexual stimuli. Task-irrelevant instructions (pull or push the stimuli according to image orientation –horizontal vs. vertical–).	Problematic Pornography Use Scale (PPUS) Brief Pornography Screen (BPS)	(1) The correlation between scores in the BPS and the approach bias score was positive and significant (*r* = 0.26). Thus, participants scoring higher in the BPS (i.e., experiencing more problems to control their pornography use) showed stronger approach biases toward sexual stimuli. (2) Participants classified as problematic pornography users demonstrated stronger approach biases toward sexual stimuli than non-problematic pornography users (*p* < .05). In particular, problematic pornography users showed more than a 200% stronger approach bias compared to individuals without this condition.
Sklenarik, Potenza, Gola, and Astur (2020)	USA	121 undergraduate females self-identified as pornography users (*M*age = 18.9; *SD* = 1.1).	Decision-making (in particular, approach-avoidance tendencies)	Approach-Avoidance Task (AAT) including both neutral and sexual stimuli. Task-irrelevant instructions (pull or push the stimuli according to image orientation –horizontal vs. vertical–).	Problematic Pornography Use Scale (PPUS) Brief Pornography Screen (BPS) Snaith-Hamilton Pleasure Scale (SHAPS) Revised Social Anhedonia Scale- Short Form (R-SAS)	(1) The correlation between scores in the PPUS and the approach bias score was positive and significant (*r* = 0.19). Thus, participants scoring higher in the PPUS (i.e., experiencing more problems to control their pornography use) showed stronger approach biases toward sexual stimuli.
Kahveci et al. (2020)	Netherlands	62 male university students (*M*age = 24.47; *SD* = 6.42): (a) 57 healthy pornography users and (b) 5 problematic users.	Decision-making (in particular, approach-avoidance tendencies)	Approach-Avoidance Task (AAT) including female stimuli (both clothed and nude).Task-relevant instructions (pull or push the stimuli according to their content –clothed vs. nude–).	Problematic Pornography Use Scale (PPUS). *Ad hoc* scale measuring frequency and intensity of pornography use.	(1) Participants reporting using pornography on a more regular basis showed stronger approach biases towards sexual stimuli (*p* = .02). However, severity of pornography consumption (measured through the PPUS) did not significantly correlate with approach bias (*p* = .81). (2) Problematic and non-problematic pornography users did not differ in terms of approach biases toward sexual stimuli (*p* = .46).

Note: Studies reviewed in this table are sorted by the cognitive domain assessed (first criterion) and the year of publication of the study in ascending order (second criterion)

The following two recorded variables (i.e., the *cognitive domain assessed* in the study and the *experimental tasks or paradigms employed* in its assessment) constituted central aspects of this review. In order to categorize studies according to the cognitive domain, we followed the taxonomy proposed by Ioannidis et al., 2019, Brand et al., 2020. In particular, we distinguished between the following cognitive domains (and subprocesses): (a) attentional bias; (b) inhibitory control (pre-potent motor inhibitory control, motor inhibitory control, and attentional inhibitory control); (c) working memory; and (d) decision making (delay discounting, approach-avoidance tendencies, and decision making under ambiguity). Then, we described the experimental paradigm used to assess these cognitive domains (type of task, stimuli employed, instructions).

In order to provide a more nuanced overview of the reviewed studies, we also recorded the use of *additional assessment measures* (interviews, self-report scales, neurological or psychophysiological measures, etc.). The last variable coded in Table 1 comprised the main findings derived from each study. Data extraction and categorizing took place in the following ways. Initially, all results derived from each study were identified from the results and conclusions sections and tabulated in text format. Subsequently, an in-depth analysis was performed to identify findings relevant to the study aims. These findings were included in table 1, whereas information beyond the scope of this review was excluded.

## 3 Results

### 3.1 Study characteristics

Table 1 summarizes studies included in the review. As for date of publication, more than half of reviewed studies (66.66%; *n* = 14) were published in the last five years. Studies were carried out in six countries and three continents: Europe (57.14%; *n* = 12), North America (23.80%; *n* = 5), and Asia (19.04%; *n* = 4).

In terms of sample size and representativeness, the studies included in this review assessed a total of 1,706 participants. Participant’s distribution for sex and age was far from being equivalent: only 26.20% of participants were females (*n* = 447), and 15 studies (71.42%) only assessed male participants. Most studies assessed participants below 30 years old (*M*age = 25.15). In terms of sexual orientation, 12 studies (57.14%) only assessed heterosexual participants. As for the sample characteristics, 52.38% of the studies (*n* = 11) reported the assessment of clinical samples, including a total of 226 patients diagnosed with PPU.

For the cognitive domains that the studies focused on, 42.85% (*n* = 9) explored decision-making, 23.80% (*n* = 5) attentional bias, 19.04% (*n* = 4) inhibitory control, and 14.28% (*n* = 3) working memory. Regarding the use of complementary assessment measures, 76.19% of the studies (*n* = 16) administered self-report scales to screen for the presence of PPU or symptoms of SA, HD, or CSBD, 38.09% (*n* = 8) included measures of other sexual dispositions (e.g., sexual excitation/inhibition), 28.57% (*n* = 6) measured impulsivity, and 19.04% (*n* = 4) used self-reports to explore psychiatric symptoms.

### 3.2 Attentional bias

Attentional bias is defined as “*the tendency for some stimuli to be preferentially processed, therefore capturing attention*” (Kagerer et al., 2014). This preconscious process explains priority when processing competing stimuli: given that our attentional resources are limited, stimuli with a greater salience are preferentially processed. This is the case of stimuli that are relevant for the survival of species (e.g., stimuli indicating a potential threat). As proposed by evolutionary models of human attention (Yorzinski, Penkunas, Platt, & Coss, 2014), this attentional bias is biologically predisposed: thus, everybody shares this predisposition. However, individual differences in the salience of certain stimuli have also been observed, influencing the allocation of attention among competing stimuli. This is a phenomenon extensively studied in SUDs (Field, Marhe, & Franken, 2014). A tendency to preferentially process drug-related cues have been documented for multiple substances (Cox, Fadardi, & Pothos, 2006). These studies show that people with SUDs notice and attend to substance-related stimuli more readily than non-substance users, and that addiction-related cues prevails over other stimuli. More recently, attentional bias toward addiction-related stimuli have been shown in different BAs, such as gambling (Hønsi et al., 2013), gaming, or problematic social networks use (Wegmann & Brand, 2020). The incentive sensitization theory have been employed to explain underlying attentional bias toward addiction-related cues (Robinson & Berridge, 2001). According to this theory, classical conditioning processes explain that addiction-cues end up eliciting attentional biases: in particular, repeated pairings of certain addiction cues with the effects derived from the drug consumption lead to an increase in the salience of these stimuli, thus ‘grabbing’ attention and becoming especially attractive and ‘wanted’.

The most popular paradigm to assess these preconscious attentional biases is the dot-probe task (van Rooijen, Ploeger, & Kret, 2017). In this task, two stimuli (e.g., words, pictures, faces) are simultaneously presented for a brief period (typically, <500 ms) in different locations of a computer screen. One of these stimuli is emotionally neutral (e.g., kitchen items), whereas the other comprises the stimulus supposed to elicit the attentional bias (e.g., a wine bottle in an alcohol-related dot-probe task). Immediately after these stimuli disappear, a neutral object (a ‘dot’) is presented in the space previously occupied by one of these stimuli, and participants should press a response button as soon as they perceive this object. Attentional bias is measured through reaction times: participants are thought to respond quickly when the ‘dot’ appears next to the stimulus they were viewing (i.e. the stimuli grabbing attention at a preconscious level). In our review, four studies employed the dot-probe task to assess attentional bias in PPU. Two of these studies used a very similar experimental design (neutral vs. sexual stimuli and 500 ms of stimuli presentation) (Doornwaard et al., 2014, Kagerer et al., 2014), whereas the other two employed a more complex design (inclusion of three types of stimuli [explicit, erotic, and neutral] and 150 ms of stimuli presentation) (Banca et al., 2016, Mechelmans et al., 2014). One study assessed attentional bias trough a different experimental paradigm (i.e., visual probe task; Pekal, Laier, Snagowski, Stark, & Brand, 2018), and two studies included complementary tasks to assess other aspects of attentional bias: a word search task measuring selective attention (Doornwaard et al., 2014) and a line orientation task measuring stimuli categorization (Kagerer et al., 2014).

Findings derived from all the reviewed studies suggest that individuals with PPU, with a greater pornography consumption, or with traits related to PPU are more likely to present attentional bias toward sexual stimuli. In a sample of 46 men and 41 heterosexual women, Kagerer et al. (2014) found that sexual sensation seekers tended to answer faster to the dot-probe task when the dot appeared next to a sex picture, and to categorize faster the pictures depicting sex in the line-orientation task. Doornwaard et al. (2014) found that participants who consumed pornography on a more regular basis (moderate and high pornography users vs. low pornography users) were faster answering to the dot probe task, independently of whether the dot appeared next to a neutral or a sexual picture. In a study comparing 22 patients with CSBD (PPU as their primary sexual problem) and 44 healthy controls, the former displayed greater attentional bias to explicit sexual stimuli (Mechelmans et al., 2014). Notably, this attentional bias was observed only when participants were presented with sexually explicit stimuli; when presented with an erotic stimulus (i.e., lower level of explicitness) or a neutral stimulus, participants with CSBD and healthy volunteers responded similarly. Reanalyzing data from this study, Banca et al. (2016) found that subjects having a greater preference for conditioned sexual stimuli (mainly, those with CSBD and PPU) also showed enhanced attentional bias for sexual stimuli. In contrast, the preference for novel vs. familiar stimuli was not associated with attentional bias for sexual stimuli. Therefore, they concluded that attentional bias toward sexual stimuli was associated with a greater preference for cues conditioned to sexual images, but not with novelty preference. This conclusion resonates with the incentive sensitization theory (Robinson & Berridge, 2001), proposing that attentional bias toward drug stimuli are the result of classical conditioning processes; however, it goes against findings from the study by Kagerer et al. (2014), which found a relationship between attentional bias and sexual sensation seeking (aka novelty preference). Finally, Pekal et al. (2018) found that attentional bias toward sexual stimuli was correlated with severity of pornography addiction, craving (i.e., desire to masturbate when presented with pornography), and subjective sexual arousal. These findings were consistent in both males and females, and partially mediated by craving and subjective sexual arousal (i.e., the effect of attentional bias upon pornography addiction was boosted by cue-reactivity and craving).

### 3.3 Inhibitory control

Inhibitory control plays a central role when it comes to regulating human behavior as it is considered responsible for suppressing thoughts, actions, and emotions in response to environmental demands: when a certain behavior is no longer relevant or is harmful (especially in the latter case), inhibitory control allows to stop and replace it with an alternative –more adapted– behavior (Verbruggen & Logan, 2008). Deficient inhibitory control is often found in multiple psychiatric conditions, including SUDs (Bechara, 2005) and BAs (Brand et al., 2016, 2019). Experimental studies have identified three levels of inhibitory control (Chamberlain and Sahakian, 2007, Howard et al., 2014): (a) motor inhibitory control (i.e., the ability to withhold not-already-triggered responses); (b) pre-potent motor inhibitory control (i.e., the ability to suppress already-triggered responses); and (c) attentional inhibitory control (i.e., the ability to suppress irrelevant cognitive processing and shift attention away from salient yet irrelevant features of the situation).

Motor inhibitory control is typically measured through the go/no-go paradigm. In this task, subjects are presented with a series of stimuli and instructed to respond as quickly as possible when a ‘go stimulus’ is presented, and to withhold their response when a ‘no-go stimulus’ is presented (e.g., “*press the response button when a horizontal line appears on the screen”* and *“do not press the response button when a vertical line appears on the screen*”). In this task, impaired response inhibition is measured through the number of omissions (participants fail to respond in a ‘go trial’) and commissions (participants fail to inhibit response in a ‘no-go trial’). In our review, only one study employed this task to explore the relationship between PPU and motor inhibitory control (Seok & Sohn, 2020). In this study, participants (30 men meeting criteria for the diagnosis of HD and a notable weekly pornography use vs. 30 healthy men reporting a moderate pornography use) completed an adapted version of this task in which neutral stimuli (letters) were presented in a neutral or sexual background. Authors found that patients with HD and an increased weekly pornography consumption performed worse in the go/no-go task than healthy controls, especially in ‘no-go trials’ (those requiring inhibition) and when the task was presented together with sexual images in the background. Therefore, they concluded that patients with HD seem to be more prone to experience problems with motor response inhibition, especially when inhibition should occur during the exposition to sexual cues.

The most popular paradigm to measure pre-potent motor inhibitory control is the stop-signal task. In a stop-signal task, subjects typically perform a choice reaction task (e.g., “*press ‘R’ after the presentation of a red circle and ‘B’ after the presentation of a blue circle*”). During certain trials (i.e., ‘stop signal trials’), subjects are presented with a stop signal after the presentation of the stimuli (e.g., an auditory signal) indicating that they should inhibit the already initiated response to the stimuli. In this task, pre-potent motor response inhibition is measured through the number of commission errors and the stop-signal reaction time (i.e., an estimate of the time taken to suppress a response that would normally be made) (Verbruggen & Logan, 2008). In our review, only one study assessed pre-potent motor inhibitory control in PPU (Antons & Brand, 2020). This research found that severity of Internet pornography use (measured through the S-IATporn –a scale assessing addiction symptoms–) and craving (i.e., strong desire to use pornography) correlated with reaction times during ‘stop-signal trials’ in both the neutral and pornographic conditions. Surprisingly, increased severity of Internet pornography use and craving was associated with faster reaction times (i.e., better pre-potent motor inhibitory control). Authors explained these contradictory findings by suggesting that subjects with a higher severity of Internet pornography use and craving may have developed certain tolerance towards pornography, meaning that the exposition to these contents was less interfering.

Attention inhibitory control is typically measured through the classical Stroop paradigm. In this task, participants are instructed to name the font color of different colored words. Participants are encouraged to respond as quickly as possible, while response time and errors are measured as outcome measures. The font color of the colored word may be congruent (e.g., the word ‘BLUE’ in blue font) or incongruent (i.e., the word ‘BLUE’ in red font), and subjects typically present delayed reaction times and increased errors in the latter condition. Attention inhibitory control is computed as the difference between subjects’ performance in congruent and incongruent conditions. In this review, only one study employed this paradigm to assess attention inhibitory control in a sample of patients with PPU meeting criteria for the diagnosis of HD (Seok & Sohn, 2018). This study found that individuals with HD and healthy controls showed similar reaction times when answering to a stroop task, but the former were less accurate when answering to incongruent stroop trials. These findings should be considered as preliminary, but they point out that patients with HD may experience certain problems to shift attention away from irrelevant stimuli. Future studies should address whether these problems are increased when using sexual stimuli as distractors.

### 3.4 Working memory

Working memory is necessary to keep things in mind while performing complex tasks, such as reasoning, comprehension, or learning (Baddeley, 2010). It is defined as “*a system for temporary storage and a mechanism for the ‘on-line’ manipulation of stored information that occurs during a wide variety of cognitive activities*” (Owen et al., 1998, p. 567) and involves two central components: a memory component (limited to events occurring in a short period of time –and sometimes equated to the concept of ‘short-term memory store’–) and a working component (necessary for understanding, problem solving, and decision making) (Cowan, 2014). At a practical level, individuals with better working memory are more efficient when it comes to integrating the analysis of current environmental information/demands with past experiences; on the contrary, individuals with working memory deficits often neglect past experiences when making present decisions, giving in to the urge of engaging in appetitive behaviors without considering potential negative consequences. As a result, working memory impairments increase the risk to engage in multiple problematic behaviors, including SUDs (Khurana, Romer, Betancourt, & Hurt, 2017) and BAs (Ioannidis et al., 2019).

The *n*-back task is one of the most popular paradigms to assess working memory (Owen, McMillan, Laird, & Bullmore, 2005). In this task, participants are instructed to monitor a series of stimuli (e.g., words or pictures) and to respond whenever a new stimulus is presented that is the same as the one presented *n* trials before. Cognitive demand required to conduct this task increases as a function of the *n* trials required to be remembered: tasks in which participants are required to respond to stimuli presented two (2-back) or three trials earlier (3-back) are considered complex. Subjects should indicate whether each stimulus was previously presented or not, and working memory is assessed by reaction times and response accuracy (Meule, 2017). In this review, we found three studies using a *n*-back task to measure working memory in PPU. Experimental tasks used to assess this cognitive domain greatly varied between studies: Sinke, Engel, Veit, Hartmann, Hillemacher, Kneer, and Kruger (2020) compared performance on a 1-back and a 2-back task while participants were presented with a neutral or a pornographic background; Au and Tang (2019) used a 3-back task after the induction of positive, negative, sexual, or neutral emotional states; and Laier, Schulte, and Brand (2013) conducted a 4-back task including pornographic pictures as stimuli. Despite these notable differences, results were highly consistent: participants with a greater pornography use and/or patients with PPU (two independent but related categories) tend to perform worse in tasks assessing working memory, especially when this cognitive domain is assessed during the presentation of concurrent sexual stimuli. Laier et al. (2013) found that subjective sexual arousal after seeing pornography and craving for porn (two basic features of PPU) correlated with different indicators of poor working memory performance. Furthermore, the interaction between these two variables predicted 27% of the variance in the performance of the 4-back task. Au and Tang (2019) confirmed that pornography users with greater problems of sexual compulsivity performed worse in working memory (less precision and increased time to answer), independently of the emotional context and the type of stimuli employed in the *n*-back test. Finally, Sinke et al. (2020) found that patients with CSBD performed worse than healthy controls when the *n*-back test was conducted with a sexual picture in the background, but not when the task was conducted with a neutral picture in the background. Notably, this study found that sexually compulsive patients performed better than healthy controls in a task measuring long-term recognition of sexual stimuli, suggesting that patients with PPU may have a better memorization/recall of sexual cues despite short-term problems with working memory.

### 3.5 Decision making

Decision making constitutes one of the most central cognitive processes as it influences on multiple aspects of goal-oriented behavior. In brief, decision making is defined as the ability to select optimal choices considering all available information (Ioannidis et al., 2019). Individuals with decision making impairments tend to show preference for short-term small gains rather than long-term large gains, experience approach tendencies toward appetitive stimuli (e.g., drugs) despite medium or long-term negative consequences, are more likely to select risky options, tend to be inaccurate when judging the probability and magnitude of potential outcomes, and tend to perseverate in their responses despite the negative results. Multiple studies demonstrate that these features are typical of individuals with SUDs (Bechara, 2005, Ernst and Paulus, 2005) and BAs (e.g., Internet gaming disorder; Schiebener & Brand, 2017), constituting the ‘core’ cognitive underpinnings of some of their self-regulation problems.

As delineated by recent theoretical models, decision making occurs on different steps comprising functionally distinct cognitive subprocesses (Ernst & Paulus, 2005). The first step of decision making (i.e., assessment and formation of preferences among the possible options) is influenced by the preference for small immediate rewards rather than large delayed rewards (i.e., discounting). Discounting is assessed by discounting tasks. These tasks measure “*the extent to which an individual devalues a reinforcer as a function of the delay to or probability of receiving it*” (Lawyer, 2008, p. 36). In a classical ‘delay discounting task’, participants are presented with a situation in which they must make a choice (e.g., “*do you want 1€ now or 10€ tomorrow?*”). In the first trials, participants typically choose delayed larger gains. Over the course of the experiment, the smaller immediate amount increases systematically (1€, 2€, 3€…) and, at some point (e.g., 8€ now or 10€ tomorrow), individuals tend to switch to the immediate outcome over the delayed outcome. In a ‘probability discounting task’, the likelihood of receiving certain outcomes changes over the course of the experiment (e.g., “*do you prefer 1€ for sure or 10€ with a 25% chance?*”). In this review, two studies used these tasks to assess discounting in PPU. One study measured delay and probability discounting for both money and erotica (Lawyer, 2008), whereas the other only measured delay discounting for money (Negash, Van, Sheppard, Lambert, & Fincham, 2016). Lawyer (2008) found that in both the monetary and erotica delay discounting tasks, erotica users preferred smaller reinforcers available immediately than larger reinforcers provided after some delay. Similarly, erotica users preferred small but certain outcomes rather than larger but uncertain outcomes. Further, the degree in which sexual behavior was problematic correlated with discounting. All in all, erotica users (especially, those showing more symptoms of PPU) tended to show more impulsive choice patterns than non-erotica users. Similarly, Negash et al. (2016) found that frequency of pornography consumption measured in time 1 predicted delay discounting four weeks later: again, participants reporting viewing more pornography demonstrated higher discounting of future rewards. Furthermore, they found that after refraining from pornography consumption for 21 days, participants reported reduced levels of delay discounting (i.e., showed an increase in their preferences for delayed longer gains). This suggests that decision making impairments related to PPU may constitute temporal deficits derived from persistent pornography use, and exercising self-control over pornography use may have a medium-term positive effect on this cognitive ability.

The first step of decision making is also influenced by another cognitive process: approach bias toward appetitive stimuli. Approach bias is defined as “*an automatically activated action tendency to approach reward-related cues*” (Kahveci, van Bocstaele, Blechert, & Wiers, 2020, p. 2). The most popular paradigm to assess this aspect is the approach-avoidance task (AAT). In the AAT, participants use a joystick to pull certain stimuli presented on a computer screen toward themselves (approach bias) or to push away (avoidance bias). The use of a joystick (i.e., physical movement) and the inclusion of a zooming feature (i.e., visual movement) enhance the effect of approaching/avoiding the stimuli. In the case of PPU, studies have focused on approach bias toward sexual stimuli: in particular, four studies used an AAT to explore the link between approach bias toward sexual stimuli and PPU. Studies varied in terms of the stimuli employed and the type of instructions provided to participants. As for the stimuli, three studies included both neutral and sexual stimuli (in particular, pictures), whereas the fourth study only included sexual stimuli. As for the task instructions, two studies used ‘task-irrelevant instructions’ (pull or push the stimuli according to image orientation –horizontal vs. vertical–) (Sklenarik et al., 2019, 2020) and two used ‘task-relevant instructions’ (pull or push the stimuli according to their content –sexual vs. neutral or clothed vs. nude–) (Kahveci et al., 2020, Snagowski and Brand, 2015). These differences may explain some of the inconsistent results found in these studies. In a study including 123 male pornography users, Snagowski and Brand (2015) found a curvilinear relationship between approach-avoidance tendencies and severity of pornography consumption: in particular, individuals with PPU showed either extreme approach or extreme avoidance tendencies toward pornographic stimuli. On the contrary, the series of studies conducted by Sklenarik et al. suggested that, both in males (2019) and females (2020), the severity of pornography consumption showed a linear (not a curvilinear) relationship with approach bias toward sexual stimuli. Furthermore, in males but not in females, individuals with PPU demonstrated stronger approach bias toward sexual stimuli than non-problematic pornography users: in particular, problematic pornography users showed more than a 200% stronger approach bias than individuals without PPU. Finally, Kahveci et al. (2020) found that individuals reporting using pornography on a more regular basis showed stronger approach biases towards sexual stimuli; however, severity of pornography consumption (measured through the Problematic Pornography Use Scale –PPUS–) did not significantly correlate with approach bias, and problematic and non-problematic pornography users did not differ in terms of approach biases toward sexual stimuli. These findings suggest that frequency –but not severity– of pornography consumption may constitute the key factor when predicting approach biases toward sexual stimuli.

The second step of decision making refers to the selection and execution of an action (Ernst & Paulus, 2005). In this step, the appraisal of risk, reward magnitudes, and the probability of different outcomes constitutes a central feature of decision making. These aspects may be assessed under two conditions: objective risk and ambiguous risk (Schiebener & Brand, 2017). Given that no studies have assessed decision making ‘under objective risk’ in PPU, we will focus on decision making ‘under ambiguous risk’. In these tasks, individuals are not provided with explicit information about the probabilities for positive/negative consequences derived from their choices before starting the task; thus, they should base their first decisions on ‘feelings’ and, over the course of the task, they can learn the implicit rules behind each decision through periodical feedback (i.e., contingency-reversal learning) (Bechara, Damasio, Tranel, & Damasio, 2005). The most popular task to assess this aspect is the Iowa Gambling Test (IGT). In the IGT, participants are given 2000€ with the indication that they should maximize their benefits over the course of the task. Participants choose cards from four decks lying face down: decks A and B are disadvantageous (high gains but even larger losses), whereas decks C and D are advantageous (moderate gains and small losses) (Buelow & Suhr, 2009). Choosing cards from decks A/B leads to overall losses, whereas cards from decks C/D leads to overall gains. Therefore, people with appropriate decision-making abilities tend to preferentially select cards from decks C/D (Steingroever, Wetzels, Horstmann, Neumann, & Wagenmakers, 2013). In this review, we found two studies measuring decision making under ambiguity through the IGT. Mulhauser et al. (2014) used a classical version of the IGT to compare decision making in a sample of 18 patients with HD (PPU as the primary sexual problem) and 44 healthy controls. These researchers found that hypersexual patients were more likely to select decks with frequent loss penalties, a pattern of response that leads to a poor performance on the IGT. Laier, Pawlikowski, and Brand (2014) employed a modified version of the IGT in which two types of stimuli (neutral vs. pornographic pictures) were alternatively assigned to the advantageous or disadvantageous desk. They assessed a sample of non-problematic pornography users, finding that performance on the IGT was better when sexual stimuli were associated with advantageous decisions and worse when associated with disadvantageous decisions (i.e., sexual cues conditioned decision making). This effect was moderated by individuals’ reactivity to pornographic contents: in individuals reporting high sexual arousal after sexual picture presentation, the influence of sexual stimuli on decision making was greater. In summary, these two studies suggest that individuals exhibiting higher reactivity in front of sexual stimuli or with PPU exhibit poor decision-making, especially when this process is guided by sexual cues. This may explain why these individuals experience problems to control their sexual behavior despite the wide range of negative consequences related to their pornography consumption.

## 4 Discussion

In the current paper, we review and compile the evidence derived from 21 studies investigating the cognitive processes underlying PPU. In brief, PPU is related to: (a) attentional biases toward sexual stimuli, (b) deficient inhibitory control (in particular, to problems with motor response inhibition and to shift attention away from irrelevant stimuli), (c) worse performance in tasks assessing working memory, and (d) decision making impairments (in particular, to preferences for short-term small gains rather than long-term large gains, more impulsive choice patterns than non-erotica users, approach tendencies toward sexual stimuli, and inaccuracies when judging the probability and magnitude of potential outcomes under ambiguity). Some of this findings are derived from studies in clinical samples of patients with PPU or with a diagnosis of SA/HD/CSBD and PPU as their primary sexual problem (e.g., Mulhauser et al., 2014, Sklenarik et al., 2019), suggesting that these distorted cognitive processes may constitute ‘sensitive’ indicators of PPU. Other studies found that these impairments in cognitive processes may be useful to distinguish between very different pornography use profiles, such as pornography users vs. non-users (e.g., Lawyer, 2008) or low pornography users vs. moderate/high pornography users (e.g., Doornwaard et al., 2014). However, other studies also found that these biases correlated with non-pathological indicators of pornography use (e.g., frequency of pornography use) (e.g., Negash et al., 2016) or with indicators of PPU in non-clinical samples (e.g., Schiebener, Laier, & Brand, 2015), suggesting that these processes may not be ‘specific’ indicators of PPU. This calls into question their usefulness to distinguish between high but unproblematic involvement and PPU, an issue that was not tested by the reviewed studies and warrants further research.

At a theoretical level, the results of this review support the relevance of the main cognitive components of the I-PACE model (Brand et al., 2016, Sklenarik et al., 2019). However, studies are inconsistent when it comes to point out ‘under which conditions’ cognitive deficits influence on PPU. Some studies found that individuals with PPU experience poor performance on different cognitive processes irrespective of the kind of stimuli used in its assessment (e.g., Au and Tang, 2019, Lawyer, 2008), suggesting that cognitive deficits are ‘stimuli-nonspecific’ and constitute a predisposition to developing self-regulation problems (in general). Other studies found that cognitive impairments appear primarily when individuals with PPU are presented with sexual stimuli (e.g., Mechelmans et al., 2014, Seok and Sohn, 2020), suggesting that cognitive deficits may be ‘stimuli-specific’ and constitute a vulnerability factor to develop sexual problems (in particular). Finally, other studies found that cognitive impairments only appear after the induction of high states of sexual arousal (e.g., Macapagal, Janssen, Fridberg, Finn, & Heiman, 2011); similarly, arousability in front of sexual contents seems to boost the link between cognitive impairments and PPU (e.g., Laier et al., 2014, Pekal et al., 2018). These last findings resonate with the concept of ‘cognitive abeyance’ proposed by the Sexhavior Cycle (Walton et al., 2017). According to this model, cognitive abeyance appears during heightened states of sexual arousal and refers to “*a state of inactivity, deferment, suspension, or diminution of logical cognitive processing*” (Walton et al., 2017). Thus, it is also possible that cognitive deficits showed in the revised studies constitute ‘transient cognitive states’ derived from PPU, and not stable predispositions. Supporting this hypothesis, Negash et al. (2016) found that refraining from pornography consumption for 21 days resulted in an increase in the preferences for delayed longer gains (i.e., a reduction of delay discounting). Therefore, the determination of the conditions under cognitive impairments in PPU appear warrants further research.

At a clinical level, in this review we have identified certain cognitive bias that are directly or indirectly linked to pathological and dysfunctional pornography use. In a recent work, Brand et al. (2020) elaborate on the difference between processes and symptoms: they state that altered cognitive processes may constitute an underlying basis for developing and maintaining symptoms of BAs (in particular, gaming disorder), but this does not mean that these processes may be useful for diagnosing this condition. According to this proposal, symptoms of PPU may be considered as behavioral and mental manifestations of the disorder and are useful for the diagnosis of this condition; in contrast, impaired cognitive processes may have limited validity as diagnostic markers but constitute important targets when developing new therapeutic approaches to PPU. In this regard, therapeutic interventions aimed to improve different executive functions have shown promising results in preventing or reducing symptoms of different SUDs (Lechner, Sidhu, Kittaneh, & Anand, 2019), and may also assist in mitigating symptoms and impact of PPU.

The studies reviewed in the current paper offer a comprehensive overview of the current state of knowledge regarding the cognitive deficits underlying PPU. However, several limitations have been identified. First, most of the participants in the reviewed studies were young heterosexual males (57.1% of the studies did not assess homosexual and bisexual participants and only 26.20% of subjects [*n* = 447] were females). Given that sex and sexual orientation modulate the manifestation of PPU (Kohut et al., 2020), evidence derived from this review should be critically appraised when generalized to females and homosexuals/bisexuals. Second, experimental tasks measuring different cognitive domains notably varied, which brings into question the comparability between studies’ results. Third, few studies assessed cognitive deficits in clinical populations, hindering the identification of clear links between these aspects and PPU. Fourth, some of the reviewed studies (mainly, those comprising patients with SA/HD/CSBD) not only included patients with PPU, but also with other out-of-control sexual behaviors. This is the way in that PPU is expressed in natural contexts (i.e., typically comorbid with other sexual problems); even when we tried to control this potential bias by eliminating studies not assessing a majority of patients with PPU as a primary sexual problem, more research is needed in order to isolate which particular cognitive processes are relevant for explaining PPU from those important for explaining out-of-control sexual behaviors in general. Similarly, many of the reviewed studies linked a certain cognitive process with a non-pathological indicator of PPU (e.g., frequency of pornography use) rather than with a direct indicator of this condition. As recent studies demonstrate that some of these ‘indirect’ indicators are not appropriate for identifying PPU (Bőthe et al., 2020), we cannot ensure that a high correlation with a certain cognitive process may be translated into an increased vulnerability to this condition. What is more, we caution against the interpretation of the findings derived from these studies as evidence of an undeniable relationship between cognitive processes and PPU. Similarly, studies conducted in non-clinical samples (an important proportion of the studies included in this review) may provide interesting findings for the topic of this review, but should not be used for drawing definitive conclusions on the relationship between cognitive processes and PPU. Finally, we acknowledge that the reviewed studies are highly heterogeneous. At this step, we considered that a comprehensive approach was warranted in order to provide a more general overview of the current state of knowledge; however, this heterogeneity may also hinder the generalizability of our conclusions. These limitations to a certain degree obscure the interpretation of findings derived from this review. Nonetheless, they also point to new and promising challenges that presumably will increase our understanding of the cognitive processes related to PPU.

Funding sources

Researchers did not receive funding for conducting this study.

Authors’ contribution

JCC and VCC were involved in the literature review, study selection, data extraction, and writing up the manuscript. RBA and CGG provided feedback on the review methodology and revised the initial draft of the manuscript. All authors read and approved the final manuscript.

## Declaration of Competing Interest

The authors declare that they have no known competing financial interests or personal relationships that could have appeared to influence the work reported in this paper.

## Footnotes

1 In this review, we use the term Behavioral Addictions (BAs) to encompass a wide variety of non-substance related addictive behaviors sharing similar features of excessive engagement, loss-of-control, and persistence despite negative consequences (e.g., pathological gambling, gaming disorder, problematic social network use, etc.). Although this construct became legitimized with the release of the DSM-5 (including gambling in the category of ‘substance use and addictive disorders’) and the ICD-11 (recognizing gambling and gaming disorder in a specific category of ‘disorders due to addictive behaviors’), the nosological status of many of the problems falling into this category is still controversial (Billieux & Maurage, 2021). Furthermore, the term BAs do not constitute a specific diagnostic category and behaviors considered potentially addictive do not represent a comprehensive and homogeneous group (Starcevic, 2016a).
